# The Potential Role of SP-G and PLUNC in Tumor Pathogenesis and Wound Healing in the Human Larynx †

**DOI:** 10.3390/biomedicines13051240

**Published:** 2025-05-20

**Authors:** Aurelius Scheer, Lars Bräuer, Markus Eckstein, Heinrich Iro, Friedrich Paulsen, Fabian Garreis, Martin Schicht, Antoniu-Oreste Gostian

**Affiliations:** 1 Institute of Functional and Clinical Anatomy, Friedrich-Alexander-Universität Erlangen-Nürnberg (FAU), 91054 Erlangen, Germany; lars.braeuer@fau.de (L.B.); friedrich.paulsen@fau.de (F.P.); fabian.garreis@fau.de (F.G.); martin.schicht@fau.de (M.S.); 2 Institute of Pathology, University Hospital Erlangen, Friedrich-Alexander-Universität Erlangen-Nürnberg (FAU), 91054 Erlangen, Germany; markus.eckstein@uk-erlangen.de; 3 Department of Otorhinolaryngology–Head & Neck Surgery, University Hospital Erlangen, Friedrich-Alexander-Universität Erlangen-Nürnberg (FAU), 91054 Erlangen, Germany; heinrich.iro@uk-erlangen.de (H.I.); antoniu-oreste.gostian@uk-erlangen.de (A.-O.G.)

**Keywords:** surfactant protein, larynx, vocal cords, squamous cell carcinoma of head and neck, wound healing, SP-G, PLUNC

## Abstract

**Background:** Immunological and rheological properties are important factors of the surfactant protein (SP) family, whose impact on tumorigenesis is not yet known, although some SPs have been identified as tumor marker candidates for various malignancies. This study describes the detection of the two surfactant family proteins SP-G and PLUNC in healthy glottis, the presence of SP-G in glottic cancer, and the in vitro tissue regeneration potential of SP-G and PLUNC on epithelial cells. **Methods:** The expression and distribution of SP-G and PLUNC were investigated immunohistochemically in squamous cell carcinomas of the vocal folds. The expression of both proteins was analyzed by Western blot in micro-dissected healthy vocal fold mucosa from body donors. The hypopharyngeal squamous carcinoma cell line (FaDu) was used as an in vitro model for wound healing experiments with Electric cell–substrate impedance sensing (ECIS). **Results:** The results show the presence of SP-G and PLUNC in epithelial cells of the healthy vocal folds and the submucosal glands of the vestibular folds. SP-G was detected in squamous cell carcinomas of the vocal folds. SP-G and PLUNC show accelerated wound healing of FaDu cells in vitro. **Conclusions:** SP-G and PLUNC were first detected in the vocal fold of the human larynx. SP-G shows a distinct presence in glottic carcinoma, whose relevance needs to be determined in future studies. SP-G and PLUNC exhibit a positive influence on the repair mechanisms of epithelial lesions of the glottis. The data presented form the basis for follow-up studies focusing on the impact of SP-G in glottic cancer development and the potentially meaningful clinical effect of SP-G and PLUNC on tissue repair of the human vocal fold.

## 1. Introduction

**Figure 1:**
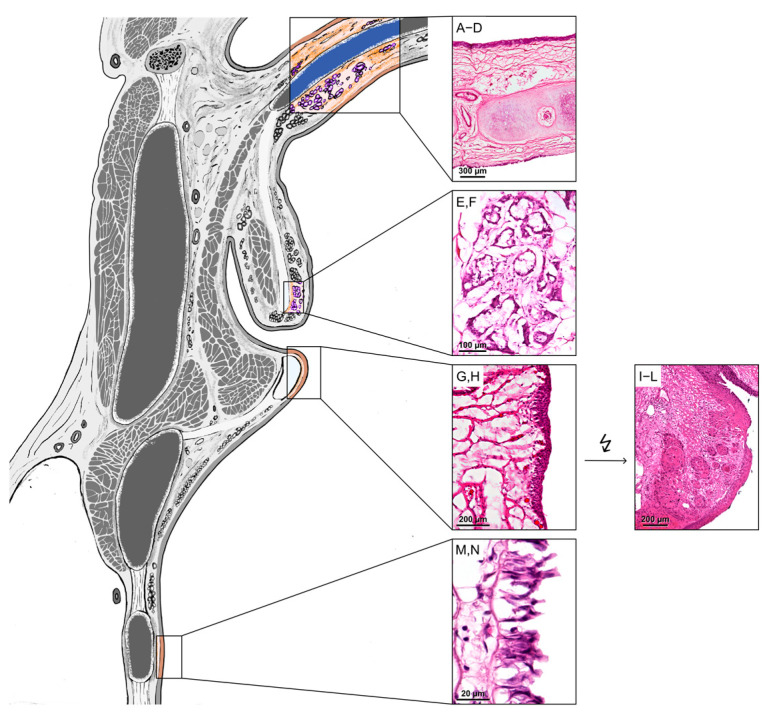
Overview of the human larynx with areas of interest for this project. (**A**–**D**): Epiglottis: oral squamous cell epithelium, laryngeal respiratory epithelium. (**E**,**F**): Vestibular fold glands: mucous-secreting glands. (**G**,**H**): Glottis: squamous cell epithelium with underlying Reinke’s space. (**I**–**L**): Glottis SCC: poorly differentiated SCC with nuclear pleomorphism and mitotic activity. (**M**,**N**): Trachea: respiratory epithelium. The lightning bolt symbolizes the dedifferentiation of healthy glottic epithelium into SCC. The labeling corresponds to the immunohistochemistry images in Figure 2.

The human larynx is a complex anatomy with multiple functions, i.e., proper breathing and swallowing, prevention of aspiration, and production of speech [1]. The vocal folds in particular play a major role in these functions: they are brought into various positions for breathing, swallowing, and speaking by their intrinsic muscles [2] and are part of the glottis (Figure 1) [3].

In Germany, larynx carcinoma is the third most common head and neck cancer (HNC), accounting for 20% of all HNC, making it the 22nd most common malignancy [4]. Around 95% of laryngeal cancers are squamous cell carcinomas (SCCs), of which approximately two-thirds are located in the glottis [5]. The most important risk factors for developing a larynx carcinoma are alcohol and smoking [6]. Larynx carcinoma patients commonly present with recently acquired hoarseness, dyspnea, dysphonia, and dysphagia. Based on the stage of the disease, primary treatment often includes surgical resection of the tumor [7]. This procedure leaves behind a wound of the glottis and adjacent parts of the larynx due to the extent of the resection, which has a definite impact postoperatively, not only on voice quality but also on proper breathing and swallowing [1], depending on the necessary resection. Thus, common complications of partial laryngectomy for laryngeal carcinoma include dyspnea and swallowing difficulties [8].

Sheats, Schroder [9] have shown that surfactant proteins A–D are inherent components of the laryngeal mucosa: they have been found in the epiglottis, vestibular folds, vocal folds, subglottis, and trachea. As for their probable functions, contribution to the innate immune defense and to the vocal fold’s mucus rheology has been discussed. SP-G and PLUNC are two novel surfactant proteins that both have been recently detected in several tissues.

SP-G (gene name: SFTA2) has so far been detected in, e.g., human lungs, eyelids, conjunctivae, lacrimal glands, kidneys, and testes [10]. It was recently found to have wound-healing properties in cultured human corneal cells [11].

PLUNC has been detected in the epithelium and glands of the upper airways and in the nasal, tonsil, and tongue glands [12] as well as in the tissue of the lacrimal system [13]. It has also been shown to be a multifunctional surface-active protein in the upper airways [14].

Furthermore, associations of a higher expression of PLUNC and SP-G mRNA with non-small-cell lung carcinoma (NSCLC) have been described [12,15]. As for SP-G, disruptions of surfactant homeostasis, e.g., by gain-of-function mutations, were suggested to contribute to the development of pulmonary diseases such as lung cancer [16]. Furthermore, an epithelial–mesenchymal transition cluster analysis of NSCLC revealed that a cluster with lower expression of SP-G was associated with elevated immune checkpoints and poorer prognosis [15]. PLUNC has also recently been proposed to downregulate the expression of the PD-L1 immune checkpoint in nasopharyngeal cancer, making it a potential candidate for checkpoint inhibition [17]. However, further investigation on the role of these proteins in tumorigenesis is necessary because, as of now, the underlying molecular pathways are still mostly unclear.

Regarding the human larynx, it is unknown until now whether SP-G and PLUNC are part of the human larynx, and what function these proteins could have in this area of the human body. We hypothesized that SP-G and PLUNC, which have already been associated with other SCCs, could also play a role in laryngeal carcinogenesis and serve as potential biomarkers or therapeutic targets. Furthermore, they could also contribute significantly to tissue regeneration and wound healing of the vocal cords. The aim of this study, therefore, was to analyze whether SP-G and PLUNC are present in healthy glottis as well as in glottic cancer. Furthermore, regarding the function of these proteins, we wanted to gain insights into the tissue regeneration potential of SP-G and PLUNC on laryngeal epithelial cells with in vitro experiments.

## 2. Materials and Methods

### 2.1. Tissue Samples

For detection, localization, and quantification of PLUNC and SP-G mRNA and protein in the three compartments (supraglottis, glottis, subglottis) of the human larynx, tissue samples were obtained from 6 body donors (4 men, 2 women, age range: 71–90 years) donated to the Institute of Functional and Clinical Anatomy (FAU Erlangen-Nuremberg, Erlangen, Germany) and 15 patients from the Department of Otorhinolaryngology of the University Hospital Erlangen whose characteristics are shown in Table 1. The study was conducted according to the Declaration of Helsinki and approved by the ethics committee of the University of Erlangen-Nuremberg (application no. 18-425_2-B; date of approval: 6 March 2023).

For protein and mRNA analysis, the vocal fold’s mucosa of the body donor’s larynges was dissected with a ZEISS OPMI PENTERO 800 surgical microscope (Carl Zeiss Meditec AG, Jena, Deutschland) and immediately frozen at −80 °C. As for the patient samples, the pathological tissue samples were already embedded in paraffin and used as such, while the healthy samples were freshly obtained and used for cryosections.

### 2.2. Cell Culture

For this study, FaDu cells from the American Type Culture Collection (HTB-43; Manassas, VA, USA) isolated from squamous cell carcinoma of a human hypopharynx [18] were acquired. FaDu cells were used, as no immortalized human vocal fold epithelial cell line was available at the time of our experiments. The cells were cultured as a monolayer with Eagle’s Minimum Essential Medium (EMEM; American Type Culture Collection, Manassas, VA, USA) supplemented with 10% fetal calf serum (FCS, Thermo Fisher Scientific Inc., Waltham, MA, USA) in a humidified incubator containing 5% CO_2_ at 37 °C. For stimulation experiments, cells (500 × 10^3^) were seeded in 6-well cell culture plates (9.4 cm^2^) and cultured until 80% confluence. Cells were washed with phosphate-buffered saline (PBS) and changed to serum-free medium for 8 h. Then, cells were either treated with bombesin, serotonin, or cortisol (Merck KGaA, Darmstadt, Germany), each at 10 µM and 0.1 µM for 12 h, 24 h, or 48 h. All experimental procedures were performed under normoxic conditions. After completion of each experiment, cells were collected and stored at −80 °C until they were processed for RNA extraction and ELISA analysis. For in vitro simulation of cell damage and subsequent wound healing, a scratch experiment was conducted using confluent FaDu cells cultured in 6-well cell culture plates (9.4 cm^2^). The confluent monolayer was scratched four times with a sterile 100 µL pipette tip, washed with PBS, and incubated with serum-free medium for different time spans (0, 15 min, 30 min, 1 h, 3 h, 6 h, 12 h, and 24 h). All experimental procedures were performed under normoxic conditions. After completion of each experiment, cells were collected and stored at −80 °C until they were processed for RNA extraction.

### 2.3. mRNA Extraction and cDNA Synthesis

mRNA was extracted from cell cultures using RNA-Solv^®^ Reagent (Omega Bio-tek Inc., Norcross, GA, USA). After centrifugation (15 min, 12,000× *g*), the supernatant was used for mRNA isolation. DNA contamination was removed by incubating the supernatant in RNase-free DNase (Thermo Fisher Scientific Inc., Waltham, MA, USA) at 37 °C for 30 min according to the manufacturer’s protocol. Reverse transcription of mRNA samples into first-strand cDNA was performed using the RevertAid Reverse Transcriptase Kit (Thermo Fisher Scientific Inc., Waltham, MA, USA) according to the manufacturer’s protocol. A total of 2 µg of total RNA was used for each reaction to cDNA. Gene-specific intron-spanning primers previously synthesized at MWG Biotech AG (Ebersberg, Germany) were used for PCR.

### 2.4. Polymerase Chain Reaction (PCR)

RT-PCR was performed according to the following standard protocol. Primers used for conventional PCR: SP-G sense (5′-TTG AGA TCT TGC ATG GTG GA-3′) and antisense (5′-CCA GCT TCC TGG AAT TGC T-3′), PLUNC sense (5′-CCC ATT CAA GGT CTT GTG GA-3′) and antisense (5′-CTG TAG TCC GTG GAT CAG CA-3′). To estimate the amount of amplified PCR product, β-actin PCR was performed with specific primers: sense (5′-GAT CCT CAC CGA GCG CGG CTA CA-3′) and antisense (5′-GCG GAT GTC CAC GTC ACA CTT CA-3′). The reference gene β-actin was used as an internal control for assessing the integrity and stability of the transcribed cDNA.

### 2.5. Quantitative Real-Time PCR

Gene expression was analyzed with quantitative Real-Time RT-PCR (qPCR) using a LightCycler480^®^ system (Roche Diagnostics International AG, Rotkreuz, Switzerland). For detection of SFTA2, the PCR mixture contained 10 µL LightCycler480^®^ 5x probe master mix, 0.25 µL of each primer, 0.4 µL Universal Probe Library probe #25 for SFTA2 or #22 for 18S (10 µM), 2 µL of each cDNA, and 7.1 µL nuclease-free water. On each 96-well plate, qPCR was performed with a cycle of 5 min at 95 °C, 55 cycles of 15 s at 95 °C, 30 s at 60 °C, and 1 s at 72 °C, to confirm amplification of specific transcripts. For detection of PLUNC, the PCR mixture contained 10 µL SYBR Green master mix, 0.25 µL of each primer, 2 µL of each cDNA, and 7.5 µL nuclease-free water. On each 96-well plate, qPCR was performed with a cycle of 5 min at 95 °C, 55 cycles of 10 s at 95 °C, 10 s at 60 °C, and 10 s at 72 °C, to confirm amplification of specific transcripts. SP-G and PLUNC primers, as well as the corresponding UPL probes (see above), were generated by using the ProbeFinder^TM^ software (Version 2.04, Roche Diagnostics International AG, Rotkreuz, Switzerland). A standard curve was generated by serial dilutions of cDNA from non-stimulated cells. To standardize mRNA concentration, the transcript levels of the housekeeping gene small ribosomal subunit (18S rRNA) were determined in parallel for each sample, and relative transcript levels were corrected by normalization based on the 18S rRNA transcript levels. Each sample was performed in duplicate, and the changes in gene expression were calculated by applying the ΔΔCt method. The primers were as follows: SFTA2 (sense 5′-TTG AGA TCT TGC ATG GTG GA-3′ and antisense 5′-CCA GCT TCC TGG AAT TGC T-3′), PLUNC (5′-CGG AGA GAA GGC AAC TGT GT-3′ and antisense 5′-CAT CAA GGT CTA AGC CTT CCA-3′), and 18S (sense 5′-GGT GCA TGG CCG TTC TTA-3′ and antisense 5′-TGC CAG AGT CTC GTT CGT TA-3′).

### 2.6. In Vitro Wound Healing Assay Using Electric Cell–Substrate Impedance Sensing (ECIS)

ECIS technology makes it possible to quantify cell morphology and migration as well as wound healing. In an ECIS experiment, cells are cultured in wells with a gold electrode (0.049 mm^2^) on the bottom of each well. Cell impedance—a property that increases with cell confluence [19]—is measured between this electrode and a larger electrode [20]. The ECIS system allows for in vitro wound healing assays by electrically creating a wound within the cell monolayer via an AC current. Wounding kills the cells on the gold electrode, thus creating a wound in the cell monolayer that results in an abrupt resistance drop [21].

For wound healing experiments, the ECIS System from Applied Biophysics Inc. (Troy, NY, USA) with suitable 8-well cell culture slides with one electrode each well and a maximum volume of 400 µL (8W1E, Ibidi GmbH, Gräfelfing, Germany) was used. Cells (120 × 10^3^) were seeded in each well of the 8W1E-slides and cultured as a monolayer until confluence. Cells were washed with phosphate-buffered saline (PBS) and changed to serum-free medium for 2 h. Then, cells were either treated with PLUNC (5 ng/mL, 100 ng/mL) or SP-G (5 ng/mL, 100 ng/mL), both from BIOZOL Diagnostica Vertrieb GmbH, Eching, Germany. After incubation for 1 h, a wound is created in each well by applying an AC current (30 s; 1600 µA; 60 kHz). After 24 h and 48 h, wounding is repeated. All experimental procedures were performed under normoxic conditions. Cell impedance was measured throughout the whole experiment at 4000 Hz. Then, the normalized impedances of the second and third woundings of each data set were combined into a mean impedance. The first wounding was excluded.

### 2.7. Immunohistochemistry (IHC) and Immunocytochemistry (ICC)

Intracellular localization of SP-G and PLUNC in human epiglottis, glottis, and trachea was performed via immunofluorescence. The analyzed areas are shown in Figure 1. For human tissue, paraffin sections (5 µm) and cryosections (5 µm) were used. The paraffin sections were deparaffinized for IHC, and epitope retrieval was performed on them. For enzymatic antigen retrieval, sections were incubated with trypsin (Merck KGaA, Darmstadt, Germany) for 10 min at 37 °C. The cryosections were thawed for 5 min and rehydrated in distilled water. IHC was performed according to a standard protocol. For SP-G and PLUNC, sections were incubated with primary antibodies (Table 2) against SP-G or PLUNC overnight at 4 °C. Then, secondary fluorescein isothiocyanate (FITC)-conjugated antibodies (Alexa 488: green, Table 2) were incubated for at least 2 h at room temperature.

For immunofluorescence analysis of FaDu cells, cells were cultured on glass coverslips and fixed with −20 °C acetone (Karl Roth, Karlsruhe, Germany) for 1 min. Cells were incubated overnight with a primary antibody mix (rabbit anti-SP-G + mouse anti-Calreticulin or rabbit anti-PLUNC + mouse anti-Calreticulin, Table 2) and for 1 h with fluorescein isothiocyanate (FITC)-conjugated antibodies (anti-rabbit Alexa 488, green; anti-mouse Alexa 555, Table 2).

Primary and secondary antibodies listed in Table 2 were used. TBST was used as a diluent.

Both sections and cells were embedded with DAPI-glycerol (PBS-glycerol 1:1, by adding 10 μL of a 2 mg/mL stock DAPI solution) on glass slides. The slides were examined using a Keyence Biorevo BZ-X800 microscope (Keyence Corporation, Osaka, Japan).

### 2.8. Western Blot

Protein was isolated from the dissected vocal fold’s mucosa using Triton X-100 lysis buffer (Thermo Fisher Scientific Inc., Waltham, MA, USA) with added protease and phosphatase inhibitors (each 10 µL/mL). Western blot was used to detect the presence of SP-G and PLUNC in proteins isolated from the mucosa of the human glottis. The sample was mixed with a protein loading buffer containing dithiothreitol as a reducing agent and heated at 98 °C for 5 min before loading. Considering the molecular weight of the samples, a 15% SDS-polyacrylamide gel for electrophoresis was chosen to separate the protein. Afterwards, separated proteins were transferred into a nitrocellulose membrane using a Semi-Dry Transfer Cell (Bio-Rad Laboratories Inc., Hercules, CA, USA). After blocking with 5% BSA in 1x PBST, the membrane was incubated with the primary antibody (Table 2) at room temperature for 1 h and afterwards at 4 °C overnight. The secondary antibody (Table 2) was incubated at room temperature for 2 h. Antibodies were diluted in 1% BSA in PBST. The bands were detected with ECL Western blotting detection reagents (EMD Millipore Corporation, Burlington, MA, USA). The molecular weights of the detected protein bands were estimated by using a standard protein marker, the Page Ruler Plus Prestained protein Ladder (Thermo Fisher Scientific, Waltham, MA, USA; ranging 10–250 kDa).

### 2.9. Enzyme-Linked Immunosorbent Assay (ELISA)

Protein was isolated from cell cultures using Triton X-100 lysis buffer (Thermo Fisher Scientific, Waltham, MA, USA) with added protease and phosphatase inhibitors (each 10 µL/mL). Quantitative sandwich ELISA was used to quantify the amount of SP-G and PLUNC in cultured cells stimulated with serotonin, cortisol, and bombesin (each 10 µM) for 24 h. For SP-G and PLUNC detection, the ELISA Kit for Surfactant Associated Protein G (SED755Hu; cloud-clone Corporation, Houston, TX, USA) and Human PLUNC ELISA Kit (RAB1173-1KT; Merck KGaA, Darmstadt, Germany) with the respective protocols were used. Quantification was accomplished by comparison with a 2.5-fold standard dilution series of the specific antigen, ranging from 0 ng/mL to 10 ng/mL. Subsequently, each sample was approximated to ng/mg.

### 2.10. Statistical Analysis

Calculations and visualizations were performed using Graph Pad Prism 10 (Graph Pad Software, San Diego, CA, USA). All data are expressed as mean ± standard error of the mean (SEM). The statistical analysis of all data except ECIS data was carried out by using a two-sided Mann–Whitney U-test, and for multiple comparisons, the Kruskal–Wallis test for nonparametric data or two-sided Welch’s *t*-test, and ordinary one-way ANOVA for parametric data, respectively. For ECIS analysis, nonlinear regression was used to fit the data into an exponential plateau model ($y=y_{max}-(y_{max}-y_{0})*e^{-k*x}$). The rate constants k of the resulting growth curves were compared by means of ordinary one-way ANOVA. Significance value was defined at * *p* < 0.05, ** *p* < 0.01, *** *p* < 0.001, or **** *p* < 0.0001 and “ns = not significant” for *p* > 0.05. Significant values are marked with asterisks as indicated above.

## 3. Results

### 3.1. Localization of SP-G, PLUNC Within the Tissue of the Human Larynx and in Cultured FaDu Cells

**Figure 2:**
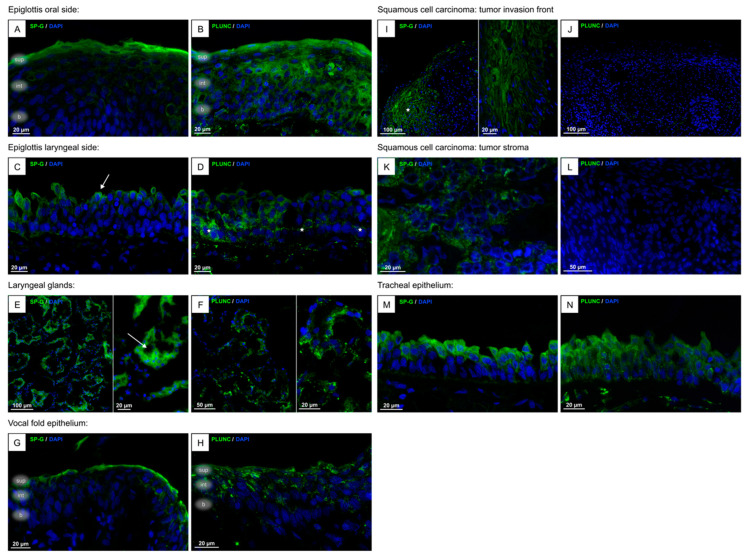
Immunofluorescence of human laryngeal tissues. Epiglottis oral side (**A**,**B**): stratum superficiale (“sup”), intermediale (“int”), and basale (“b”) can be distinguished, SP-G reactivity was found mainly in the superficial cell layer. PLUNC reactivity was found in all cells; epiglottis laryngeal side (**C**,**D**): SP-G reactivity at apical poles of the ciliated cells (arrow). Signal for PLUNC predominantly in the basal cells (asterisks), but also in the ciliated cells; vestibular fold glands (**E**,**F**): reactivity for SP-G and PLUNC antibody in the acinar cells, for PLUNC predominantly at the apical poles. Arrow shows secretion of SP-G into the lumen; vocal fold epithelium (**G**,**H**); strong signal for SP-G in stratum superficiale (“sup”), faint signal in stratum intermedium (“int”), almost no signal in stratum basale. PLUNC reactivity with similar intensity across all epithelial cells; squamous cell carcinoma (SCC) of the vocal fold ((**I**,**J**): tumor invasion front, (**K**,**L**): tumor stroma): the cancer cells of the tumor invasion front (asterisk in **I**) and tumor stroma (**K**) show strong reactivity for the SP-G-antibody. No signal for PLUNC; tracheal epithelium (**M**,**N**): signal for SP-G only in the ciliated cells. PLUNC reactivity in basal and ciliated cells. (SP-G/PLUNC: green; DAPI (4′,6-diamidino-2-phenylindole) = blue).

**Figure 3:**
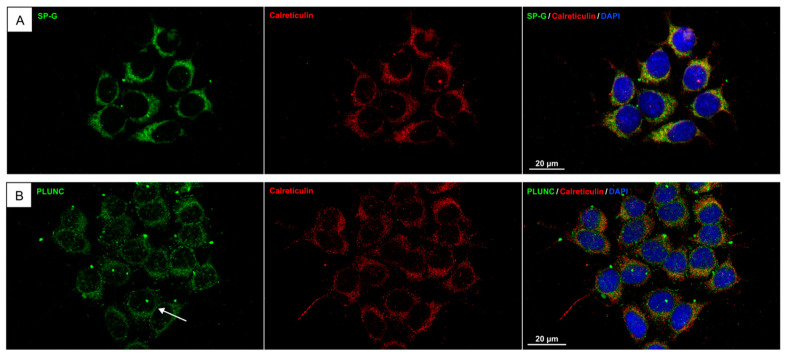
Immunofluorescence of human hypopharyngeal cell line (FaDu): demonstration of antibody reactivity for SP-G (**A**) and PLUNC (**B**). Double staining with calreticulin showed a coincidence of SP-G/PLUNC and calreticulin signals. The arrow in B shows small lines of PLUNC-positive reactivity at the cell membranes. (SP-G/PLUNC: green; calreticulin: red; DAPI (4′,6-diamidino-2-phenylindole) = blue).

SP-G and PLUNC were detected in all healthy laryngeal tissue samples as well as in FaDu cells (Figure 2 and Figure 3). The green fluorescence signal represents a positive antibody reaction against SP-G or PLUNC in tissue samples and FaDu cells. The red fluorescence signal represents a positive antibody reaction against calreticulin. While cryosections were used for healthy tissue, for SCC samples, only paraffin-embedded sections were available. Samples incubated with secondary antibody showed no reactivity.

In detail, the epiglottis’ oral side (Figure 2A,B): Positive antibody reaction with SP-G was found mainly in the uppermost superficial cell layer. The signal for SP-G decreases in the lower layers of the squamous cell epithelium. PLUNC reactivity was found in all cells of the oral side of the epiglottic epithelium.

Epiglottis laryngeal side (Figure 2C,D): SP-G reactivity could be detected especially in the apical poles of the ciliated cells (arrow), but not in the basal cells. A signal for PLUNC, on the other hand, was found predominantly in the basal cells of the respiratory epithelium (asterisks), but also in the ciliated cells.

Vestibular fold glands (Figure 2E,F): Acinar cells show a reaction with the SP-G and PLUNC antibody. Secretion of SP-G into the lumen could be visualized (arrow). The PLUNC reactivity is stronger at the apical poles of the acinar cells. A weak signal of intraluminal PLUNC reactivity could be shown.

Vocal fold epithelium (Figure 2G,H): The squamous cell epithelium shows a stronger signal for SP-G in the stratum superficiale and a faint signal in the stratum intermedium. In the stratum basale, almost no signal is found. PLUNC reactivity was found with similar intensity across all vocal fold epithelial cells.

Vocal fold SCC (Figure 2I–L): The cancer cells of both the tumor invasion front (asterisk in Figure 2I) and the tumor stroma (Figure 2K) show a strong reactivity for the SP-G-antibody. The SCC cells did not show a signal for PLUNC (Figure 2J,L).

Tracheal epithelium (Figure 2M,N): Comparable to the laryngeal side of the epiglottis, a signal for SP-G was only in the ciliated cells and not in the basal cells. PLUNC reactivity was found in all tracheal epithelial cells.

FaDu cells (Figure 3): Immunocytochemistry of FaDu cells demonstrated that both SP-G and PLUNC are distributed in the cytoplasm and perinuclear region. Double staining with calreticulin revealed a coincidence of SP-G and calreticulin signal, suggesting an intracellular location of SP-G at the endoplasmic reticulum (ER). The PLUNC signal also showed a coincidence with the calreticulin signal. In addition to this, small lines of PLUNC-positive reactivity could be seen at the cell membranes (arrow in Figure 3B), suggesting that PLUNC is a cell-membrane-associated protein in laryngeal mucosal cells.

Thus, our analysis shows that healthy glottis epithelial cells and FaDu cells express SP-G and PLUNC.

### 3.2. Detection of SP-G, PLUNC in the Human Vocal Fold Mucosa and in Cultured FaDu Cells

**Figure 4:**
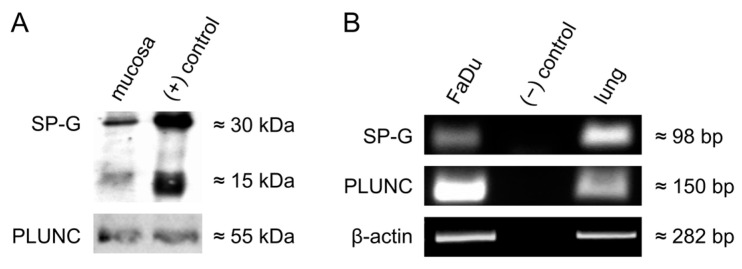
(**A**): Western blot analysis of SP-G and PLUNC in micro-dissected human vocal fold mucosa. Protein isolated from human bronchoalveolar lavage was used as a positive control. (**B**): RT-PCR analysis of SP-G and PLUNC mRNA in FaDu cells. Samples without cDNA were used as negative controls, whereas cDNA isolated from human lung tissue was used as a positive control.

Protein extracts of vocal fold mucosa (n = 4) were tested for the presence of SP-G and PLUNC by WB analysis, which revealed distinct protein bands for PLUNC and SP-G, respectively (Figure 4A). For both proteins, distinct bands were found to be at the expected height (PLUNC: 55 kDa; SP-G: 15 kDa). SP-G was also found to be at 30 kDa for all analyzed tissues. Bronchoalveolar lavage served as a positive control for SP-G and PLUNC.

RT-PCR analysis of FaDu cells revealed the presence of SP-G and PLUNC mRNA (Figure 4B). The β-actin control PCR was positive for all samples. The detected PCR bands were in accordance with the expected sequences from PLUNC and SP-G within the NCBI gene bank data (https://www.ncbi.nlm.nih.gov/genbank, accessed on 5 May 2025).

### 3.3. Effects of Bombesin, Cortisol, Serotonin, and Mechanical Scratch on SP-G and PLUNC Expression in FaDu Cells

**Figure 5:**
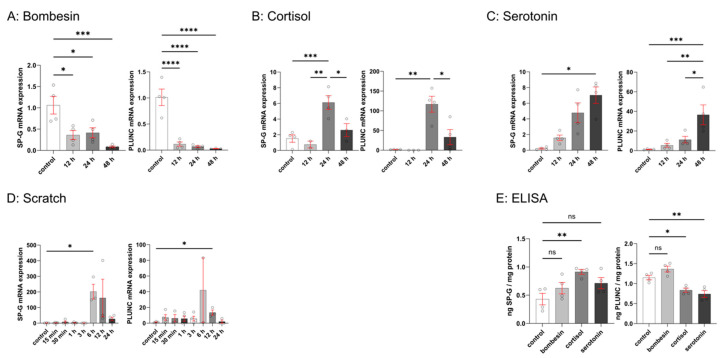
Stimulation of FaDu cells for 12, 24, and 48 h after treatment with 10 µM bombesin (**A**), cortisol (**B**), and serotonin (**C**). While stimulation with bombesin led to a significant decrease in SP-G and PLUNC mRNA, a significant increase was seen after stimulation with cortisol and serotonin. After 48 h of stimulation with cortisol, the gene expression of SP-G and PLUNC decreased again. (**D**): Simulation of wound healing of FaDu cells for 15 min, 30 min, 1 h, 3 h, 6 h, 12 h, and 24 h after mechanical scratching. Scratching led to a significant increase in SP-G and PLUNC mRNA after 6h and 12 h, respectively. Both mRNA decreased again after 24 h (**E**): stimulation of FaDu cells for 24 h after treatment with 10 µM bombesin, cortisol, and serotonin. A significant increase in cortisol was seen after stimulation with cortisol, and a significantly lower PLUNC concentration was seen after stimulation with cortisol and serotonin. SP-G and PLUNC mRNA expression of FaDu cells after stimulation was determined by qRT-PCR. SP-G and PLUNC proteins were quantified using ELISA. mRNA expression levels and protein concentration are expressed as mean ± SEM (n = 4). Significance value was defined at * *p* < 0.05, ** *p* < 0.01, *** *p* < 0.001, or **** *p* < 0.0001 and “ns = not significant” for *p* > 0.05.

The behavior of FaDu cells after stimulation with cortisol, serotonin, bombesin, and after mechanical damage through scratching was examined (Figure 5). Because the role of SP-G and PLUNC during wound healing was suspected, gene expression was also measured after creating a wound within the cell layer by qPCR. After stimulation with cortisol and serotonin, and after scratching, an increase in SP-G and PLUNC mRNA was seen. Scratching led to the highest increase in SP-G mRNA, whilst stimulation with 10 µM cortisol led to the highest increase in PLUNC mRNA. Stimulation with bombesin led to a significant decrease in both SP-G and PLUNC mRNA expression. In case of stimulation by scratching and cortisol, the SP-G and PLUNC mRNA expression decreased again after 24 h (scratching) or 48 h (cortisol). That effect was not seen after stimulation with serotonin and bombesin.

ELISA was used to verify whether induction of SP-G and PLUNC mRNA expression also led to a significant increase in intracellular protein concentration (Figure 5E). PLUNC protein concentration (1.15 ng/mg) was found to be than twice as high as SP-G protein concentration (0.43 ng/mg). In the case of SP-G, one could only see a significant increase after stimulation with cortisol, whilst with PLUNC, there was a significantly lower PLUNC concentration in the case of stimulation with cortisol and serotonin. Bombesin did not induce a significant change in protein concentration.

### 3.4. In Vitro Wound Healing Assay (ECIS)

**Figure 6:**
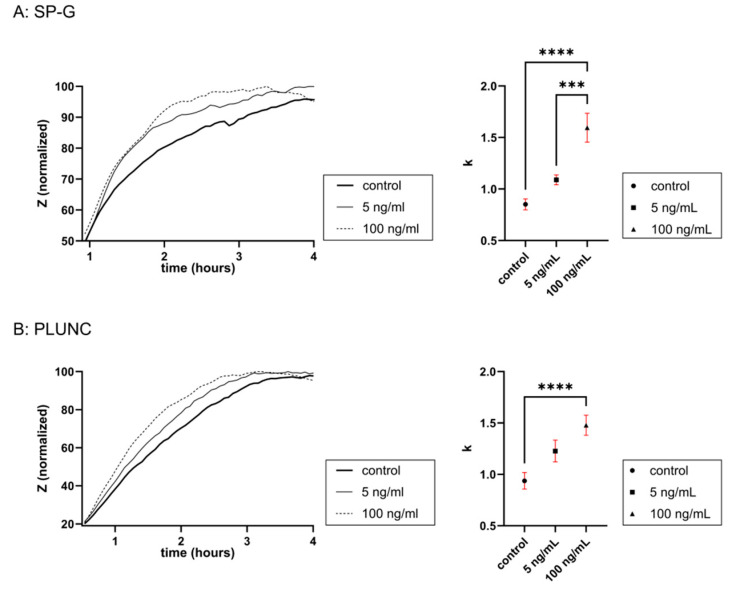
In vitro wound healing assay (ECIS). (**A**,**B**) After electrically creating a wound via an AC current, an increase in mean cell impedance in the FaDu monolayer cultures incubated with various stimulants in serum-free medium was compared to a control (serum-free medium only) (n = 8). To better visualize the data on the left graphs, the impedances were normalized so that 0 is defined as the first measurement after wounding (t = 0) and 100 is defined as the largest mean in each data set. The rate constants k of the exponential plateau model via nonlinear regression (right graph, model: $y=y_{max}-(y_{max}-y_{0})*e^{-k*x}$) correlate with the growth rate and were significantly higher during stimulation with SP-G (**A**) and 100 ng/mL PLUNC (**B**). Thus, the addition of SP-G and PLUNC correlates with a positive effect on in vitro wound healing in the FaDu cells. Significance value was defined at *** *p* < 0.001 and **** *p* < 0.0001.

The ECIS-based wound healing assay on cultured FaDu cells mimicked epithelial cell damage and demonstrated the effect of various stimulations on the wound healing rate (Figure 6). Within the first 4 h after creating the wound, stimulation with SP-G and PLUNC showed a steeper increase in impedance after wounding (t = 0) in comparison to the unstimulated control (= higher value of the rate constant *k*). That correlates with the positive effect of SP-G and PLUNC on wound healing. Stimulation with PLUNC and SP-G revealed an effect of concentration. With both proteins, the higher concentration of 100 ng/mL increased the cell impedance more than 5 ng/mL.

## 4. Discussion

SP-G was first described by Zhang and Henzel [22] as a surfactant protein encoded on human chromosome 6 that was found in several human tissues such as the lung, eyelid, conjunctiva, kidney, and testis [10] as well as in the lacrimal gland, meibomian gland, and corneal epithelium [11]. PLUNC was described as a protein of the upper airways that is encoded on chromosome 20 [23]. Later, PLUNC was recognized as a surfactant protein [24] that is also produced at the ocular surface and the lacrimal apparatus [13].

We show for the first time that both SP-G and PLUNC are expressed in the human larynx. Using immunofluorescence analysis, SP-G and PLUNC were detected in all analyzed laryngeal tissues, accounting for all anatomical sites of the larynx. Both proteins are produced by the epithelial cells of the epiglottis, glottis, and trachea as well as the acinar cells of the submucosal glands of the vestibular folds. The association of both proteins with the endoplasmic reticulum (ER) in FaDu cells suggests that they are secretory proteins synthesized in the ER. A possible secretion through epithelial cells is especially plausible in the case of SP-G because immunoreactivity could be observed in luminal cells at their apical poles. PLUNC reactivity was found in all epithelial cells. The production and secretion of SP-G and PLUNC by the squamous and acinar cells of the glottis leads to the hypothesis that they are part of the viscous mucus that covers the vocal folds. In molecular dynamics simulations, PLUNC and SP-G have been shown to have high potential to interact with phospholipid membranes [11,13], which corresponds with our findings. Other surfactant proteins, such as SP-A, SP-B, SP-C, and SP-D, have also been detected in the laryngeal mucosa and glands [9]. However, neither surfactant proteins A-D, SP-G, nor PLUNC have yet been proven to be part of the vocal fold mucus. Although the existence of surfactant proteins in the vocal fold mucus is highly suggestive, further experiments are needed to confirm that.

Furthermore, both SP-G and PLUNC proteins were detected in the human vocal fold mucosa via Western blot. In the case of SP-G, besides a distinct protein band at 15 kDa, another one at 30 kDa was seen. Considering that the protein might undergo post-translational modifications, such as phosphorylation, glycosylation, and palmitoylation, resulting in different physiochemical properties for better lipid interaction [10], the protein band at 15 kDa seems to represent the mature protein. Also, the better-known surfactant proteins A-D are known to undergo posttranslational modifications [25,26,27,28]. The protein band at 30 kDa seems to represent a dimeric form of SP-G. This expression pattern of SP-G has also been found in the immortalized alveolar type II cell line A549, human lung tissue, and bronchoalveolar lavage [10,11,29]. For the better-known surfactant proteins B [30] and C [31], it has already been described that they form dimers that are supposed to play an important role in the trafficking and functioning of these proteins.

Regarding the functions of SP-G and PLUNC in the human larynx, one can only make assumptions because little is known about these novel surfactant proteins. The human larynx is in constant interaction with many pathogens, which makes it an important organ for immunological decision-making in the airway [32]. Pathogens causing upper respiratory tract infections are mostly viral, but also bacteria such as Streptococcus pyogenes, H. influenzae, N. meningitidis, and C. diphtheriae are present [33]. PLUNC has been proposed to have an antibacterial function because it is strongly related to proteins of the BPI (bactericidal/permeability-increasing) family that are able to bind lipopolysaccharide (LPS) of Gram-negative bacteria [34]. Furthermore, PLUNC has been shown to inhibit the formation of Pseudomonas aeruginosa biofilms in in vitro experiments by Gakhar and Bartlett [24]. The function of SP-G was also linked to the immune system. For instance, Krause and Peukert [35] demonstrated that in cerebrospinal fluid (CSF), SP-G is increased in bacterial infection of the central nervous system and suggested an involvement in host defense and maintenance of rheological properties in the CSF. In the lungs of mice, SFTA2 mRNA was found to be downregulated by tracheal LPS instillation [29]. Thus, both SP-G and PLUNC were associated with Gram-negative bacteria. The presence of both proteins in the epithelium and vestibular fold glands of healthy laryngeal mucosa, and possibly being part of the laryngeal mucus, suggests that SP-G and PLUNC are potentially able to interact with bacteria that enter the upper airways. Thus, it seems reasonable to assume that SP-G and PLUNC play a role in host defense of the larynx.

As SP-G and PLUNC have been found to have surface tension-reducing properties [11,24], one can also propose a rheological function in the larynx. Studies have shown that an increased viscosity of the vocal fold’s mucus results in decreased vocal fold vibration and thus leads to decreased voice quality [36,37]. Furthermore, it was found that patients who suffer from phoniatric disorders have thicker appearing mucus aggregation [38,39]. Surface active proteins such as SP-G and PLUNC might help reduce the viscosity of the vocal fold mucus and thus improve voice quality.

In addition, they might have a beneficial impact on wound healing on the vocal folds. SP-G has already been shown to have a beneficial impact on wound healing in a cell culture model of human corneal epithelium [11].

The increased wound healing might not only be caused by surface tension reduction and, thus, a better environment for cell migration. One could also suspect that SP-G might also induce cell proliferation itself. That is indicated by the fact that SP-G was found to be expressed in human squamous cell carcinoma cells of the glottis. Furthermore, SP-G mRNA was found to be increased in NSCLC [15]. Another explanation for the association of SP-G and cancer cells might be that SP-G is upregulated because of a cancer-related inflammation of the surrounding tissue [40] and an increase in cortisol in the patients’ blood [41]. Further investigation must be performed to see if more types of cancer are associated with SP-G and whether SP-G is primarily cancer-related or a reaction to the inflammation reaction induced by the tumors. For PLUNC, a complete absence of the protein was seen in the analyzed SCC cells (Figure 2J). This corresponds to the findings of Lemaire and Millon [42], which show that PLUNC RNA is downregulated in head and neck SCC, and Bingle and Cross [12], who did not find PLUNC by means of IHC of squamous cell carcinoma. This finding is very interesting because it suggests that PLUNC is suppressed in malignant squamous cells. Due to immune evasion mechanisms by cancer as well as chronic inflammation in the cancer microenvironment, laryngeal SCC is frequently associated with upregulation of immune checkpoints such as PD-1, PD-L1, and CTLA-4 [43]. In a recent study, Feng, Guo [17] could demonstrate a negative correlation between PLUNC expression and the expression of PD-L1 in nasopharyngeal carcinoma, making it a possible candidate for immune checkpoint blockade. Regarding that, down-regulation of PLUNC in laryngeal SCC could therefore be part of a cancer-induced immune evasion mechanism. On the other hand, Bingle and Cross [12] found that PLUNC is expressed in distinct types of NSCLC, such as adenocarcinoma, muco-epidermoid carcinoma, and bronchio-alveolar carcinoma. Also, PLUNC mRNA was associated with NSCLC and proposed as a potential marker for micrometastases [44]. Furthermore, PLUNC protein was found to be a marker protein to differentiate between gastric hepatoid adenocarcinoma (PLUNC expression) and hepatocellular carcinoma (no PLUNC expression) [45]. Further investigation must be performed to understand why PLUNC is supposedly suppressed in certain types of cancer cells but not in others.

For the in vitro experiments, FaDu cells originating from a hypopharyngeal SCC were used. One can suppose that FaDu cells have similar properties to vocal fold epithelial cells because both are squamous cells. Furthermore, FaDu cells have already been implanted in the supraglottis and glottis of mice to create an orthotopic mouse model of laryngeal squamous cell carcinoma [46]. Therefore, they were used as an in vitro model of the vocal fold epithelium.

ELISA analysis of unstimulated FaDu cells revealed that PLUNC protein concentration (1.15 ng/mg) is more than twice as high as SP-G protein concentration (0.43 ng/mg). Further experiments are needed to see whether the cell culture model matches the in vivo situation. This, however, matches the hypothesis that PLUNC, which was found to make up 8–10% of the protein in secretions of cultured human tracheobronchial epithelial cells [47], has a central role as a protein in the upper respiratory system [23].

Cortisol is widely known to play an important role in lung maturation and to induce the synthesis of surfactant proteins in the lungs [48]. To examine the effects of cortisol stimulation on SP-G and PLUNC expression, concentrations of 0.1 µM and 10 µM were used. These concentrations are present within the human body after the experience of stress (0.1 µM), respectively, and after taking cortisol for therapeutic reasons (10 µM) [11]. For serotonin and bombesin, the same concentrations were used to make the effects of the different stimulants more comparable. It has already been shown by Schicht and Riedlova [11] that stimulating cultured human cornea epithelial cells with cortisol resulted in an increase in intracellular SP-G mRNA. That corresponds to our findings that stimulating FaDu cells with cortisol led to an increase in SP-G and PLUNC mRNA. However, only SP-G protein concentration increased significantly after stimulation with cortisol, while PLUNC protein concentration even decreased.

Serotonin-reactive cells have been found in human lungs from the eighth week of gestation [49]. Moreover, it has been shown that in the alveolar stage of lung development, the expression of serotonin receptors is much stronger in type II pneumocytes—responsible for the secretion of SP- A and -D—in comparison to type I pneumocytes. So, it has been proposed that type II pneumocytes play a possible role in surfactant production and secretion by Nikolić, Vukojević [49]. Stimulation of FaDu cells with serotonin led to an increase in both SP-G and PLUNC mRNA. However, no significant increase in protein concentration was found. One possible explanation for that might be a delayed protein synthesis after an increase in mRNA, which is an issue that has already been discussed both for prokaryotic [50] and eukaryotic cells [51].

Our experiments showed that the stimulation of FaDu cells with bombesin led to a significant decrease in SP-G and PLUNC mRNA. Bombesin was also found to inhibit alveolarization and thus the production of surfactant proteins [52]. On the other hand, bombesin seemingly has a dose-dependent impact on surfactant lipid secretion [53] with a maximum at 3 nM bombesin and a decrease at higher concentrations. Thus, it may be that stimulation with lower bombesin concentrations could result in a stimulatory effect on surfactant proteins.

In all wound healing assays that have been performed, the electrically created wound was closed after a maximum of 4 h. This suggests that the wound healing was mostly achieved by cell migration into the wounded area. Our study revealed that not only are SP-G and PLUNC mRNA increased after mechanical wounding of the FaDu cells, but also that the addition of both proteins to injured cells correlated with an increased wound healing rate in this cell line. This strongly supports the proposed thesis that both examined surfactant proteins, SP-G and PLUNC, could have a beneficial role in wound healing of the glottis epithelium. Wound healing effects have already been shown for SP-G [11]. In the case of PLUNC, an increase in wound healing through the addition of PLUNC protein has not been shown until now. However, Hu and Li [54] have shown, in a wound healing assay of a lung adenocarcinoma cell line, that inhibiting PLUNC mRNA via siRNA reduced the migration of the cells. Additionally, Sung and Moon [55] have shown that PLUNC is upregulated after injury of the olfactory epithelium and have suggested a possible protective function against bacterial infection in injured epithelial tissues. As our experiments were conducted in a sterile environment, the observed increase in wound healing in our experiments was not due to an antibacterial function of PLUNC, but rather the effect of an increase in cell migration.

Limitations: The number of tissue samples and their European origin can be seen as limiting. To further undermine our results, one should consider performing experiments with a higher sample size, including patients of different ethnicities. One further limitation is the hypopharyngeal squamous cell line used as an in vitro model of human vocal fold epithelium. One can, however, suppose that FaDu cells have similar properties to vocal fold epithelial cells, as stated previously. Additionally, in wound healing experiments, the influence of cell growth stimulation on the accelerated wound healing cannot be excluded, and proliferation and differentiation assays evaluating cell proliferation markers such as Ki-67 are needed.

One should perform further experiments to undermine our thesis of SP-G and PLUNC playing a significant role in the laryngeal carcinogenesis. Such a study could involve in vitro tests using human glottis epithelial cell lines to correlate the proteins with known pathways of cancer formation, e.g., by siRNA knockdown of target genes. Additionally, future research could involve the in vivo outcome in glottic wound healing or carcinogenesis, depending on the presence of SP-G or PLUNC.

## 5. Conclusions

In summary, SP-G and PLUNC have been detected in all parts of the human larynx. Both proteins have been shown to be involved in wound healing in cultured squamous epithelial cells. They might play a role during inflammatory diseases, conserving the integrity of the vocal fold’s mucus and the underlying squamous cells through a reduction in surface tension. SP-G might be associated with squamous cell neoplasia. The presented pioneer data strongly indicate that both proteins potentially have significant roles in the physiological function of the larynx, wound healing, and cancer formation of the laryngeal glottis that merit further investigations.

## Figures and Tables

**Table 1:** Characteristics of patients from the Department of Otorhinolaryngology of the University Hospital Erlangen whose samples were used in this study. The table shows patient demographics and the T category of the patients’ TMN classification.

Characteristics	n (%)
Total patients	15
Male	13 (86.7)
Female	2 (13.3)
Age (in years)	
Mean	62.6 ± 14.1
Median	68
Range	28–80
Tumor status	
T0	2 (13.3)
pT1	3 (20)
pT2	1 (6.7)
pT3	5 (33.3)
pT4	4 (26.7)

**Table 2:** Primary and secondary antibodies were used for immunofluorescence and Western blot analysis. IF = immunofluorescence. WB = Western blot.

Antibody	Method	Dilution	Company, Catalog Number
rabbit anti-SP-G	IF, WB	1:50, 1:200	cloud-clone, PAD755Hu01
rabbit anti-PLUNC	IF	1:50	santa-cruz, SC-271457
mouse anti-calreticulin	IF	1:100	Invitrogen, MA5-15382
goat anti-PLUNC	WB	1:250	santa-cruz, SC-49248
goat anti-rabbit, Alexa 488	IF	1:1000	Invitrogen, A11070
goat anti-mouse, Alexa 488	IF	1:500	Invitrogen, A11029
goat anti-rabbit, Alexa 555	IF	1:1500	Invitrogen, A21428
goat anti-mouse, Alexa 555	IF	1:500	Invitrogen, A21424
goat anti-rabbit, HRP-conjugated	WB	1:5000	Dako, P0448
donkey anti-goat, HRP-conjugated	WB	1:5000	santa-cruz, SC-2020

## Data Availability

Please contact the authors for data requests (A.S.; email: aurelius.scheer@fau.de).

## Abbreviations

The following abbreviations are used in this manuscript:

MDPI	Multidisciplinary Digital Publishing Institute
DOAJ	Directory of open access journals
BPI	Bactericidal/permeability-increasing
BSA	Bovine serum albumin
DAPI	4′,6-diamidino-2-phenylindole
cDNA	Complementary deoxyribonucleic acid
CSF	Cerebrospinal fluid
ECIS	Electric cell–substrate impedance sensing
ELISA	Enzyme-Linked Immunosorbent Assay
EMEM	Eagle’s Minimum Essential Medium
ER	Endoplasmic reticulum
FCS	Fetal calf serum
HNC	Head and neck cancer
ICC	Immunocytochemistry
IHC	Immunohistochemistry
LPS	Lipopolysaccharide
mRNA	Messenger ribonucleic acid
NCBI	National Center for Biotechnology Information
NSCLC	Non-small-cell lung cancer
PBS	Phosphate-buffered saline
PCR	Polymerase chain reaction
PD-L1	Programmed Death-Ligand 1
PLUNC	Palate, lung, and nasal epithelium clone
qPCR	Quantitative Real-Time reverse transcription polymerase chain reaction
SCC	Squamous cell carcinoma
SEM	Standard error of the mean
SP	Surfactant protein
UPL	Universal Probe Library
